# Medical surveillance unit: patient characteristics, outcome, and quality of care in Saskatchewan, Canada

**DOI:** 10.1186/s13104-020-04951-4

**Published:** 2020-02-21

**Authors:** Karl Vantomme, Muhammad Siddiqui, Marlee Cossette, Kish Lyster

**Affiliations:** 1 College of Medicine, University of Saskatchewan, Saskatoon, SK Canada; 2 Department of Research, Saskatchewan Health Authority, Regina, SK Canada; 3 Medical Surveillance Unit, Pasqua Hospital, Saskatchewan Health Authority, Regina, SK Canada

**Keywords:** Quality of care, Intermediate care unit, Patient flow, Mortality

## Abstract

**Objective:**

Intermediate care units provide a high level of care to complex patients and are becoming increasingly popular in North America. Despite the growing popularity of Intermediate care units, very little is known about them. This study explored a typical Intermediate care unit, identifying patient characteristics including demographics, comorbidities, length of stay, as well as primary and secondary diagnosis and mortality.

**Results:**

A total of 200 patients chart were reviewed, of which, 102 were male, and 89 patients were younger than 65 years old. Diabetes, hypertension, and chronic obstructive pulmonary disease were common among patients with a prevalence of 33.5%, 56%, and 32.5%, respectively. Alcohol use disorder, asthma, liver disease and IV drug abuse were much more common in patients younger than 65 years. The average length of stay was 5.31 days regardless of age. Almost two-thirds of the patients in the Intermediate care unit were admitted directly from the emergency room. The mortality rate among the patients studied was 9.5%. The most common admitting diagnosis was respiratory diseases such as chronic obstructive pulmonary disease or Pneumonia (38.0%), followed by cardiac disorders which were predominantly arrhythmias and congestive heart failure (27.0%).

## Introduction

Critically ill patients require complex care in a highly developed environment where they receive enhanced patient care. Intermediate care units (IMCUs) provide a high level of care to complex patients and are becoming increasingly popular in North America [1]. The purpose of IMCUs in hospitals is to act as a dedicated ward for patients who do not require the level of care an intensive care unit provides but need closer monitoring than a general ward [2, 3]. This lessens the burden for both the general ward and the intensive care unit, allowing more space and the appropriate level of care to be provided to patients [4–6]. The IMCU can be used as a step-down from the intensive care unit (ICU) once the patient has stabilized, but can also accommodate patients from the emergency department or post-op patients. This can facilitate faster times to inpatient beds thus shortening emergency wait times that are important markers for many hospital systems as well as decreasing pressure on scarce ICU beds [7].

The structure, efficiency, and functioning of IMCUs vary by geographical location [8]. Due to the buffer function of the IMCUs, the duration of ICU admission can be reduced and it seems reasonable that the lower need for ICU beds decreases health care costs. However, there is relatively little data published to support this benefit. Most of the literature regarding IMCU is based in the United States or Europe, so there is very little information based in Canada. Furthermore, information regarding the demographics and disease profiles of Canadian patients admitted into an IMCU is sparse [9].

The source of information for this study is an IMCU located in an urban center in Saskatchewan, a prairie province in central Canada. This IMCU has been in operation since January 15, 2016. The unit examined admits a mixture of emergency medical and surgical patients, and receives approximately 700 admissions per year. Despite the theoretical reasons to support the use of IMCUs and the many such units now in existence, there are relatively little published data directly assessing their value. The main purpose of this study was to explore typical IMCU patient characteristics including demographics, comorbidities, length of stay, primary and secondary diagnosis, and mortality.

## Main text

This study is a retrospective chart review of medical surveillance unit, Pasqua hospital, Regina. The medical charts of 200 patients admitted into the medical surveillance unit (MSU) in the Pasqua hospital in Regina were retrospectively reviewed. Each patient was assigned a unique study identification number. No unique patient identifiers such as medical record numbers were entered into the database and the database was de-identified. Data analysis was performed on a de-identified dataset in order to protect patient confidentiality.

Information was gathered by a medical student who was not involved in the assessment or treatment of the patients studied. Information regarding patients’ demographics, primary and secondary diagnoses, co-morbidities, discharge disposition, reason for hospitalization, duration of stay in hospital and in the MSU, and location of admission or transfer to the MSU were obtained. The patients’ reasons for hospitalization as well as the primary and secondary diagnoses were then sorted into categories for analysis.

This study has been reviewed and approved on ethical grounds by the research ethics board of the former Regina Qu’Appelle Health Region, Regina, SK, Canada (REB/18-38). Statistical analysis was performed using SPSS Statistics software (Version 22.0. Armonk, NY: IBM Corp.). Data was expressed in frequencies, mean and percentages. Chi square test was used as a test of significance to compare differences between groups for categorical data and *t* test/Mann–Whitney U test was used for continuous data. Significance will be set at p < 0.05 level.

The summary of the data is presented in Table 1. Of these, 102 were male, and 89 patients were younger than 65 years old. 67 of those studied were diagnosed with diabetes, and the prevalence of diabetes was similar between age groups. Approximately 15% of patients have kidney disease. 7.5% of patients have asthma, and patients younger than 65 years old were about three times as likely to have it (p < 0.02). Chronic Obstructive Pulmonary Disease was common among patients with a prevalence of 32.5%, but prevalence did not vary by age. Coronary heart disease was more than three times as likely in patients older than 65 compared to the younger age category (p < 0.001). Liver disease had a prevalence of 5%, but was 10 times as likely in the younger age category (p < 0.003). Dementia was exclusively found in the older age category with a prevalence of 4.5% (p < 0.006). 27% of the patients studied consumed cigarettes. Those in the younger age category smoke twice as much than those in the older age category (p < 0.004). Cerebrovascular disease had a prevalence of 9.5% and was seven times more likely in the older age group (p < 0.002).

**Table 1:** Selected demographic characteristics of participants—n (%)

Characteristics	Total	Age ≤ 65 years	Age > 65 years	p-value
Gender				*0.91*
Male	102 (51)	45 (50.6)	57 (51.4)	
Female	98 (49)	44 (49.4)	54 (48.6)	
Diabetes				*0.58*
No	133 (66.5)	61 (68.5)	72 (64.9)	
Yes	67 (33.5)	28 (31.5)	39 (35.1)	
Hypertension				< *0.001*
No	88 (44)	57 (64)	31 (27.9)	
Yes	112 (56)	32 (36)	80 (72.1)	
Kidney disease				*0.40*
Not applicable	171(85.5)	81(91)	90 (81.1)	
eGFR > 90	4 (2)	1 (1.1)	3 (2.7)	
eGFR 60–89	9 (4.5)	2 (2.2)	7(6.3)	
eGFR 30–59	10 (5)	4 (4.5)	6 (5.4)	
eGFR 15–29	5 (2.5)	1 (1.1)	4 (3.6)	
eGFR < 15	1 (0.5)	0	1 (0.9)	
Asthma				*0.02*
No	185 (92.5)	78 (87.6)	107 (96.4)	
Yes	15 (7.5)	11 (12.4)	4 (3.6)	
COPD				*0.37*
No	135 (67.5)	63 (70.8)	72 (64.9)	
Yes	65 (32.5)	26 (29.2)	39 (35.1)	
Coronary heart disease				*0.001*
No	168 (84)	83 (93.3)	85 (76.6)	
Yes	32 (16)	6 (6.7)	26 (23.4)	
Liver disease				*0.003*
No	190 (95)	80 (89.9)	110 (99.1)	
Yes	10 (5)	9 (10.1)	1 (0.9)	
Dementia				*0.006*
No	191 (95.5)	89 (100)	102 (91.9)	
Yes	9 (4.5)	0	9 (8.1)	
Smoking				*0.004*
No	146 (73)	56 (62.9)	90 (81.1)	
Yes	54 (27)	33 (37.1)	21 (18.9)	
Cerebrovascular disease				*0.002*
No	181 (90.5)	87 (97.8)	94 (84.7)	
Yes	19 (9.5)	2 (2.2)	17 (15.3)	
Intravenous drug users				< *0.001*
No	181 (90.5)	70 (78.7)	111 (100)	
Yes	19 (9.5)	19 (21.3)	0	
Alcohol use disorder				*0.007*
No	174 (87)	71 (79.8)	103 (92.8)	
Yes	26(13)	18 (20.2)	8 (7.2)	

Substance abuse disorders were common, 17% of patients had alcohol or drug related problems. Intravenous drug use had a prevalence of 9.5% among all the patients. Another 13% of patients had alcohol use disorder, with the younger age category nearly three times more likely to have alcohol use disorder compared to the older age category (p < 0.007) (Table 1).

**Fig. 1:**
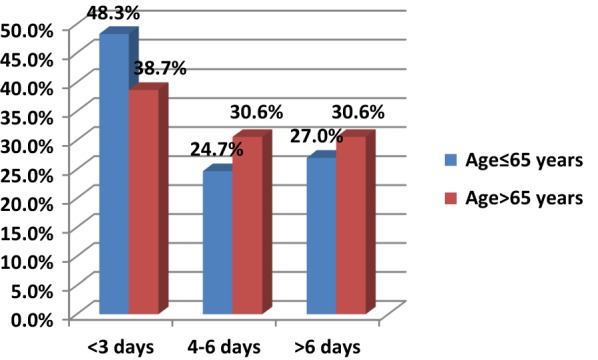
Duration of stay in medical surveillance unit

Nearly half of the younger age category spent fewer than 3 days in the MSU. The rest of the younger age category were equally likely to stay for 4–6 days or > 6 days. Over a third of the older age category spent fewer than 3 days in the MSU. Just under a third of the patients older than 65 stayed in the MSU for 4–6 days and > 6 days (Fig. 1).

The emergency room and the medical ward were the largest sources of admission or transfer to the MSU, contributing 65% and 21% of the patients, respectively. The ICU contributed 9% of the patients in the MSU while the OR contributed 5%. The average duration of stay across all age groups was 5.31 ± 5.04 days. Almost half of the patients (49%) were discharged from the MSU to a ward. Just over a third of MSU patients were transferred home (36%), 9.5% of patients were deceased and 5.5% of patients were transferred to an alternate level of care. Nearly two-third of study participants (65%) were admitted through emergency department and 21% transferred from wards (Table 2).

**Table 2:** Location of admission or transfer and duration of stay—n (%)

	< 5 days	6–15 days	> 15 days	Total
ER	39 (76.5)	59 (69.4)	32 (50)	130 (65)
ICU	1 (2)	7 (8.2)	10 (15.6)	18 (9)
OR	4 (7.8)	4 (4.7)	2 (3.1)	10 (5)
Wards	7 (13.7)	15 (17.6)	20 (31.3)	42 (21)
Total	51 (25.5)	85 (42.5)	64 (32)	200 (100)

*ER* emergency room, *ICU* intensive care unit, *OR* operation room

The most common primary diagnosis accounting for more than a third (38%) of all primary diagnoses was respiratory disease. This also accounts for almost a third of all hospital visits and was the most common reason among MSU patients for visiting the hospital (31.5%). The most common secondary diagnosis was cardiovascular disorders (27%) accounting for over a quarter of all secondary diagnoses. Substance abuse, renal disease, musculoskeletal injury, and cancer each accounted for less than 10% of all primary and secondary diagnoses as well as reason for hospital visit. Metabolic disorders account for about a fifth of all primary diagnoses (17%), secondary diagnoses (21.5%), and reason for hospital visit (19.5%).

Little information has been published concerning patient demographics and disease profiles in IMCUs. Basic demographics have been published in Europe and the United States, though these studies lack in-depth data on patients’ disease profiles [4, 6, 10]. Furthermore, the data from these studies may not be generalizable to the Canadian population. This study conducted an in-depth analysis on 200 patients in an IMCU in central Canada.

Many of the demographic measures considered in this study are similar to previous studies conducted in the United States and Europe. Similarly to Lu et al. [11] and Fernandes et al. [12], slightly more than 60% of the patients in the IMCU were transferred from the Emergency Department. This suggests that most patients use the IMCUs as a step-up unit from the ER rather than a step-down unit from the ICU.

The introduction of an IMCU contributed to a more appropriate use of ICU facilities and did result in a significant increase in mean nursing workload at the ICU [13]. Lu et al. found that the average length of stay in the IMCU was 4.23–7.24 days, which is similar to our average of 5.31 ± 5.04 days (data not shown) [11]. They found that patients transferred from the ICU had a longer length of stay in the IMCU, so it is likely that the average patient will stay longer in the IMCU if a higher proportion of patients are transferred from the ICU. However, Lu et al.’s [11] study limited analysis to only those patients that transferred from the ER and the ICU, whereas our study analyzed all patients in the IMCU. Because the majority of patients came from the Emergency Department and ICU in both studies the results are still comparable. A different study based in Portugal conducted by Fernandes et al. [12] found an average length of stay of 10.18 ± 9.07 days. This range is much more variable than Lu et al.’s and our study, but this may be due to regional and staffing differences.

This study is one of the first to analyze comorbidities among IMCU patients such as diabetes, cerebrovascular disease, and alcohol use disorder). Over 30% patients in the IMCU have diabetes and over half the patients have hypertension. These numbers are above the average prevalence in Canada [14]. The prevalence of these comorbidities is likely so high in the IMCU because they are risk factors for more serious illnesses. Fernandes et al.’s [12] study measured similar comorbidities and included pulmonary disease, kidney diseases, and metabolic diseases with a prevalence of 34.7%, 3.1%, and 5.2%, respectively. Our study measured respiratory disorders, renal disease, and metabolic disorder with a prevalence of 38.0%, 4.5%, and 17.0%, respectively. While the renal disease and respiratory disorder prevalence is similar, our metabolic disease prevalence is high. This difference may be due to regional differences or how the hospital studied treats diabetic patients. Simpson et al. found that most patients in the IMCU suffer from respiratory and cardiac disorders, which is in agreement with the results from this study [4].

An IMCU can provide care for patients who do not require intensive care support, but need a higher level of nursing care that cannot be provided on the general ward. The IMCU concept was suggested as a strategy to promote earlier discharge from ICU, facilitate patient re-allocation, decrease costs and prevent unnecessary ICU readmissions [15–17]. One of the unique characteristics of our MSU was patient discharge disposition. About half of patients are sent to a general medical ward. Interestingly, approximately 40% are discharged to home or alternative levels of care. This has significant implications to staffing for the provision of services needed to set up home care, etc. The mortality rate in our MSU was 9.5%, which is remarkably similar to Fernandes et al.’s study which found a mortality of 9.38% [12].

## Limitations

One of the main limitations of this study is that the 200 patients studied were the first 100 unique patients in 2017 and 2018. This means that all of the patients studied were admitted between January and April of each year. Certain diseases have increased rates of hospitalization depending on the time of year, so by limiting the time-frame to the early months of the year certain diseases may be misrepresented [18]. Another limitation is the retrospective nature of the data. Some conditions may have been missed because the data was not transcribed or easily found in the record.

This study did not address cost or resource utilization for the cohort of patients, and thus cannot come to any conclusions regarding the utility of the unit from a system perspective. What is clear is that most patients used the MSU as a step up in care intensity instead of a step down as some have suggested [7]. Future work might explore what this means from a system perspective.

This study is one of the first to describe these demographic data points among medical surveillance unit patients in Canada. Identification of this previously unrecognized IMCU population should act as the impetus for investigating and implementing appropriate care plans for complex patients.

## Data Availability

The datasets during and/or analysed during the current study are available from the corresponding author on reasonable request.

## Abbreviations

IMCU: Intermediate care units
ICU: Intensive care unit
MSU: Medical surveillance unit
TCPS: Tri-Council Policy Statement
